# Diabetes, use of metformin, and the risk of meningioma

**DOI:** 10.1371/journal.pone.0181089

**Published:** 2017-07-14

**Authors:** Corinna Seliger, Christoph R. Meier, Claudia Becker, Susan S. Jick, Martin Proescholdt, Ulrich Bogdahn, Peter Hau, Michael F. Leitzmann

**Affiliations:** 1 Department of Neurology and Wilhelm Sander-NeuroOncology Unit, Regensburg University Hospital, Regensburg, Germany; 2 Basel Pharmacoepidemiology Unit, Division of Clinical Pharmacy and Epidemiology, Department of Pharmaceutical Sciences, University of Basel, Basel, Switzerland; 3 Boston Collaborative Drug Surveillance Program, Boston University School of Public Health, Boston University, Boston, Massachusetts, United States of America; 4 Hospital Pharmacy, University Hospital Basel, Basel, Switzerland; 5 Department of Neurosurgery, Regensburg University Hospital, Regensburg, Germany; 6 Department of Epidemiology and Preventive Medicine, University of Regensburg, Regensburg, Germany; The University of Hong Kong, HONG KONG

## Abstract

**Background:**

Metformin is a commonly used oral antidiabetic agent that has been associated with decreased cancer risk. However, data regarding the association between metformin use and the risk of meningioma are unavailable.

**Methods:**

We conducted a matched case-control analysis using data from the U.K.-based Clinical Practice Research Datalink (CPRD) to analyse diabetes status, duration of diabetes, glycemic control, and use of metformin, sulfonylureas, and insulin in relation to the risk of meningioma. We conducted conditional logistic regression analyses to determine relative risks, estimated as odds ratios (ORs) with 95% confidence intervals (CIs) and adjusted for body mass index, smoking, history of arterial hypertension, myocardial infarction, and use of estrogens (among women).

**Results:**

We identified 2,027 meningioma cases and 20,269 controls. For diabetes there was the suggestion of an inverse association with meningioma (OR = 0.89; 95%CI = 0.74–1.07), which was driven by an inverse relation among women (OR = 0.78; 95%CI = 0.62–0.98), in whom we also noted a suggestive inverse association with duration of diabetes (p-value for trend = 0.071). For metformin there was a suggestive positive relation, particularly after matching on duration of diabetes and HbA1c level (OR = 1.64; 95%CI = 0.89–3.04). Sulfonylureas showed no clear association (OR = 0.91; 95%CI = 0.46–1.80). For insulin there was the suggestion of an inverse relation, in particular when comparing a high vs. low number of prescriptions (OR = 0.58; 95%CI = 0.18–1.83).

**Conclusion:**

Further studies are needed to solidify a possible inverse association between diabetes and meningioma risk and to clarify the role of antidiabetics in this context.

## Introduction

Meningioma is a common meningeal intracranial or intraspinal tumor, affecting about 8 patients per 100.000 person-years [1]. The incidence of meningioma increases with age, affecting men less often than women [1]. Established risk factors for meningioma are uncommon and they include a history of ionizing radiation and rare genetic cancer syndromes [2].

Female sex hormones [3, 4], adiposity [5, 6], and arterial hypertension [6, 7] may be associated with increased risk of meningioma. However, there is conflicting evidence on whether diabetes is positively related [8, 9], unrelated [10] or inversely [11, 12] related to the risk of meningioma. Metformin is a frequently prescribed oral antidiabetic agent [13], which has been associated with reduced cancer risk [14], but specific data regarding metformin use and associated meningioma risk are unavailable. Metformin inhibits the mammalian target of rapamycin (mTOR) [15]. Meningioma samples have been shown to express high levels of mTORC1, indicating mTOR signalling as a relevant pathway in meningioma development [16]. Further, inhibitors of mTORC1 reduce meningioma growth in mice [17]. However, the only study investigating treatment of meningioma cells with metformin *in vitro* showed no effects at clinically relevant doses [18].

The plausible underlying biological mechanisms and the sparse observational data regarding diabetes and use of metformin in relation to the risk of meningioma prompted us to perform the current study.

## Patients and methods

### Data source

The Clinical Practice Research Datalink (CPRD) is a primary care database in the United Kingdom (U.K.), which holds patient information on around 8.5% of the population of the U.K. Patient data in the CPRD are representative of the U.K. general population with respect to age, sex, and ethnicity. General practitioners record demographic data, physical findings, symptoms, diagnoses, referrals, hospital admissions, drug prescriptions, and deaths in an anonymous format using standard coding systems [19]. The CPRD has been extensively validated [20, 21] and found to be of high quality. The current study was reviewed and approved by the Independent Scientific Advisory Committee of the CPRD (protocol-number 16–121) and the protocol was made available to the journal reviewers.

### Study population

The study population was comprised of all people in the CPRD during years 1995 to 2015 who were age ≤90 years.

#### Case definition

We defined cases as patients in the study population who had a first ever Read code for meningioma during the indicated study time. See S1 Table for a list of Read codes used to identify cases. The index date for each case was the date of diagnosis minus three years. We did this to account for potential lag time between disease development and diagnosis, and to increase the likelihood of assessing exposure before meningioma onset to minimize bias due to early symptoms of undiagnosed meningioma, such as the earlier detection of pre-existing concomitant diseases, or changes in drug adherence and usage patterns. We excluded patients with less than three years of active history in the database before the index date, those with a current or past history of other cancers except non-melanoma skin cancer and those with recorded alcoholism or human immunodeficiency virus infection prior to the index date.

#### Control definition

We matched up to 10 controls for each case, randomly selected from the study population, on sex, age (same year of birth ±2 years), calendar time (same index date), general practice, and number of years of active history in the database prior to the index date. We applied the same exclusion criteria to controls as to cases.

### Exposures

We assessed use of metformin, sulfonylureas, and insulin before the index date for cases and controls. We categorized exposure to antidiabetic drugs, based on the number of prescriptions before the index date, into short-term use (1–9 prescriptions), medium-term use (10–29 prescriptions), or long-term use (≥ 30 prescriptions). The number of prescriptions served as an approximation of exposure duration, since an average prescription covers 45–90 days of treatment, depending on the number of tablets (1 or 2) taken per day. Exposure was assessed separately for each study drug. If more than one study drug was received, we mutually adjusted our analyses for drug use, such that results relating use of a particular antidiabetic drug to risk of meningioma were adjusted for combined or prior use of other antidiabetic drugs.

We assessed the presence of a diagnosis of diabetes mellitus, the duration of diabetes, and the mean recorded glycosylated haemoglobin A1c (HbA1c) level from the computerized records. Duration of diabetes was calculated by counting the days between the date of the first documentation of a diabetes diagnosis and the respective index date. We classified duration of diabetes into three categories (< 2 years, 2–5 years, > 5 years) for cases and controls, and HbA1c levels into four categories (unknown, <6.5%, 6.5–7.9%, ≥ 8.0%). Our analysis was not restricted to patients with type 2 diabetes. However, when we considered patients younger than 30 years of age with insulin use as an estimation of patients with type 1 diabetes, only 2 patients in our dataset were deemed type 1 diabetics.

### Statistical analysis

We conducted conditional logistic regression analysis using SAS statistical software version 9.4 (SAS Institute Inc, Cary, NC) to determine relative risks, estimated as odds ratios (ORs) with 95% confidence intervals (CIs) of meningioma in relation to diabetes status, duration of diabetes, level of glycemic control, and use of specific antidiabetic drugs. In univariate analyses, we investigated the associations of meningioma to various potential confounding variables, including presence versus absence (reference) of specific medical conditions, diseases or medications, such as dyslipidemia, stroke, transient ischemic attack (TIA), ischemic heart disease (IHD), myocardial infarction (MI), congestive heart failure (CHF), and renal failure; and use of statins, non-steroidal anti-inflammatory drugs, and aspirin. We included variables that were significantly associated with risk of meningioma in univariate analyses in the final multivariate analysis in addition to BMI, smoking status, and arterial hypertension. Analyses were also performed without adjustment for BMI to prevent statistical over control.

We conducted tests of linear trend by modeling the median value of duration of diabetes, HbA1 level, or drug prescription category as a continuous variable in the multivariate model, the coefficient for which was evaluated using a Wald test. We considered a two-sided p-value of <0.05 statistically significant.

We stratified our results by age and sex and reported the relevant differences in the text. We also performed subanalyses restricted to diabetic patients and we additionally matched on diabetes, duration of diabetes, and both duration of diabetes and HbA1c level.

## Results

We ascertained 2,027 cases and 20,269 controls in the CPRD database. Cases and matched controls had a mean age ± standard deviation (SD) of 61.6 ± 15.2 years at the index date, and most cases were women (75.7%). The mean number of years of active history in the database was 11.2 ± 5.0 years before the index date. As compared to normal weight (BMI 18.5–24.9 kg/m^2^), overweight (BMI 25–30 kg/m^2^) was suggestively related to increased risk of meningioma (OR = 1.13; 95%CI = 1.00–1.27) and obesity (BMI ≥30 kg/m^2^) was statistically significantly associated with increased risk of meningioma (OR for obesity = 1.31; 95%CI = 1.15–1.50).

Table 1 displays general characteristics of meningioma cases and controls. Cases and controls were similar with respect to most covariates. Use of 1–8 prescriptions of estrogens in women (OR = 1.39; 95%CI = 1.18–1.63) was associated with a small increased risk of meningioma, whereas the opposite was true for history of myocardial infarction (OR = 0.67; 95%CI = 0.49–0.91). No associations with meningioma were found for dyslipidemia, stroke, CHF, renal failure, and use of statins, non-steroidal-anti-inflammatory drugs, and aspirin.

**Table 1:** Characteristics of meningioma cases and controls.

Variable	Number of cases (%) (n = 2,027)	Number of controls (%) (n = 20,269)	Crude OR (95%CI)	p-value
**Age (years)**^**a**^				
0–9	1 (0.1)	10 (0.1)	-	-
10–19	17 (0.8)	173 (0.9)	-	-
20–29	24 (1.2)	261 (1.3)	-	-
30–39	117 (5.8)	1,161 (5.7)	-	-
40–49	301 (14.9)	3,018 (14.9)	-	-
50–59	396 (19.5)	3,963 (19.6)	-	-
60–69	455 (22.5)	4,526 (22.3)	-	-
70–79	461 (22.7)	4,638 (22.9)	-	-
80–90	255 (12.6)	2,519 (12.4)		
**Sex** ^**a**^				
Women	1,534 (75.7)	15,340 (75.7)	-	-
Men	493 (24.3)	4,929 (24.3)	-	-
**Comorbidities**				
Dyslipidemia	228 (11.3)	2,110 (10.4)	1.11 (0.95–1.29)	0.207
Stroke/TIA	89 (4.4)	958 (4.7)	0.92 (0.73–1.16)	0.481
CHF	43 (2.12)	449 (2.5)	0.85 (0.61–1.18)	0.326
MI	45 (2.2)	656 (3.2)	0.67 (0.49–0.91)	0.011
Renal Failure	53 (2.6)	514 (2.5)	1.03 (0.77–1.38)	0.828
**Statins**				
No prior use	1,669 (82.3)	16,753 (82.7)	1.00 (referent)	-
1–9 Rx	99 (4.9)	842 (4.2)	1.18 (0.95–1.48)	0.135
≥10 Rx	259 (12.8)	2,674 (13.2)	0.98 (0.83–1.14)	0.747
**NSAIDs**				
No prior use	528 (26.1)	5,583 (27.5)	1.00 (referent)	-
1–9 Rx	1,364 (67.3)	13,456 (66.4)	1.09 (0.97–1.22)	0.153
≥10 Rx	135 (6.7)	1,230 (6.1)	1.19 (0.96–1.46)	0.112
**Aspirin**				
No prior use	1,953 (96.4)	19,523 (96.3)	1.00 (referent)	-
1–14 Rx	57 (2.8)	533 (2.6)	1.07 (0.81–1.42)	0.647
≥15 Rx	17 (0.8)	213 (1.05)	0.79 (0.48–1.31)	0.367
**Estrogens**^b^				
No prior use	1,113 (72.6)	11,685 (76.2)	1.00 (referent)	-
1–8 Rx	241 (15.7)	1,922 (12.5)	1.39 (1.18–1.63)	<0.0001
≥9 Rx	180 (11.7)	1,733 (11.3)	1.16 (0.97–1.40)	0.105

^a^Matching variables: age, sex, general practice, and number of years of active history in the database.

^b^Women only.

BMI: Body Mass Index; TIA: transient ischemic attack; CHF: congestive heart failure; MI: myocardial infarction; NSAIDs: non-steroidal anti-inflammatory drugs; OR: odds ratio; Rx: total number of prescriptions prior to index date.

Diabetes mellitus was associated with a statistically non-significant decreased risk of meningioma (OR = 0.89, 95%CI = 0.74–1.07) (Table 2). Stratification by sex revealed an inverse relation of diabetes and risk of meningioma in women (OR = 0.78; 95%CI = 0.62–0.98) but not men (OR = 1.17; 95%CI = 0.85–1.61), whereas the relation did not vary according to age. Among the 90 diabetic women, 32 (35.6%) were using exogenous estrogens.

**Table 2:** Risk of meningioma in relation to diabetes status, duration of diabetes, and HbA1c level, overall and stratified by sex and age.

Variable	Number of cases (%) n = 2,027	Number of controls (%) n = 20,269	Adjusted OR^a^ (95%CI)
**Total meningioma**			
Diabetes	145 (7.2)	1,465 (7.2)	0.89 (0.74–1.07)
**Women**			
Diabetes	90 (5.9)	1,001 (6.5)	0.78 (0.62–0.98)
**Men**			
Diabetes	55 (11.2)	464 (9.4)	1.17 (0.85–1.61)
**<40 years**			
Diabetes	0 (0.0)	18 (1.1)	n.e.
**40–69 years**			
Diabetes	64 (5.6)	592 (5.1)	0.93 (0.70–1.23)
**>69 years**			
Diabetes	81 (11.3)	855 (12.0)	0.88 (0.68–1.29)
**Total glioma**			
**Duration of Diabetes**			
Non-diabetic	1,882 (92.9)	18,804 (92.8)	1.00 (referent)
< 2 years	24 (1.2)	229 (1.1)	0.93 (0.61–1.42)
2–5 years	32 (1.6)	375 (1.9)	0.75 (0.52–1.08)
> 5 years	89 (4.4)	861 (4.3)	0.94 (0.74–1.18)
p-value for trend			0.423
**HbA1c level**			
Unknown	1,858 (91.7)	18,700 (92.3)	1.05 (0.83–1.31)
< 6.5%	96 (4.7)	927 (4.6)	1.00 (referent)
6.5–7.9%	40 (2.0)	314 (1.6)	1.19 (0.81–1.77)
≥ 8.0%	33 (1.6)	328 (1.6)	0.97 (0.64–1.47)
p-value for trend			0.977

^a^Matched on age, sex, general practice, and number of years of active history in the database, and adjusted for BMI, smoking, arterial hypertension, MI, and use of estrogens.

The p-value for trend did not include subjects with unknown HbA1c level.

When we restricted the analysis to those women, the inverse association between diabetes and risk of meningioma was lost (OR = 1.09; 95%CI = 0.69–1.75). In the overall study population, there was no clear trend regarding increasing duration of diabetes (p-value for trend for increasing duration of diabetes = 0.423) or level of glycemic control (p-value for trend for increasing HbA1c = 0.977) and the risk of meningioma. In women however, there was a borderline significant inverse association between increasing duration of diabetes and meningioma risk (p-value for trend = 0.071). Results were similar when we removed BMI from the analysis, though the previously observed inverse association between diabetes and meningioma in women was no longer statistically significant (OR = 0.83; 95%CI = 0.66–1.05), nor was the previously observed borderline significant trend of increasing duration of diabetes and risk of meningioma in women (p-value for trend = 0.188).

Use of 10 metformin prescriptions or more was associated with a statistically non-significant increased risk of meningioma (OR for ≥30 prescriptions = 1.16; 95%CI = 0.76–1.77, Table 3). When we restricted the analysis to diabetic patients, 90 diabetic cases (62.1% of diabetic cases) and 857 diabetic controls (58.5% of diabetic controls) were taking metformin. When analysing a newly matched dataset containing diabetic patients only, use of metformin was associated with a non-significantly increased risk of meningioma (OR for use versus non-use of metformin = 1.16; 95%CI = 0.77–1.76; OR for ≥30 prescriptions = 1.27; 95%CI = 0.79–2.04). The relation strengthened after matching on duration of diabetes and HbA1c level (OR for ≥30 prescriptions = 1.64; 95%CI = 0.89–3.04, p-value for trend = 0.059). When we stratified by sex, the positive test for trend regarding the association between metformin use and risk of meningioma in analyses matched by duration of diabetes and HbA1c level was statistically significant in women (p-value for trend = 0.033).

**Table 3:** Risk of meningioma in relation to number of prescriptions for anti-diabetic drugs.

Antidiabetic drug and no. of prescriptions	Meningioma cases and controls			Diabetic meningioma cases and controls, matched on duration of diabetes			Diabetic meningioma cases and controls, matched on duration of diabetes and HbA1c level		
	Cases (%) (n = 2,027)	Controls (%) (n = 20,269)	Adjusted OR^a^ (95%CI)	Cases (%) (n = 142)	Controls (%) (n = 1,328)	Adjusted OR^a^ (95%CI)	Cases (%) (n = 125)	Controls (%) (n = 837)	Adjusted OR^a^ (95%CI)
**Metformin**									
0	1,932 (95.3)	19,395 (95.7)	1.00 (referent)	53 (37.3)	514 (38.7)	1.00 (referent)	47 (37.6)	401 (47.9)	1.00 (referent)
1–9	18 (0.9)	209 (1.0)	0.88 (0.52–1.47)	17 (12.0)	196 (14.8)	0.99 (0.54–1.80)	17 (13.6)	133 (15.9)	0.84 (0.43–1.64)
10–29	34 (1.7)	275 (1.4)	1.25 (0.83–1.89)	30 (21.1)	244 (18.4)	1.40 (0.83–2.37)	28 (22.4)	150 (17.9)	1.42 (0.78–2.58)
≥30	43 (2.1)	390 (1.9)	1.16 (0.76–1.77)	42 (29.6)	374 (28.2)	1.20 (0.69–2.08)	33 (26.4)	153 (18.3)	1.64 (0.89–3.04)
p-value for trend			0.403			0.498			0.059
**Sulfonylureas**									
0	1,962 (96.8)	19,572 (96.6)	1.00 (referent)	79 (55.6)	670 (50.5)	1.00 (referent)	73 (58.4)	534 (63.8)	1.00 (referent)
1–9	15 (0.7)	134 (0.7)	0.94 (0.53–1.69)	15 (10.6)	139 (10.5)	0.81 (0.43–1.52)	15 (12.0)	76 (9.1)	1.27 (0.65–2.45)
10–29	12 (0.6)	208 (1.03)	0.50 (0.26–0.93)	12 (8.5)	189 (14.2)	0.49 (0.25–0.96)	10 (8.0)	115 (13.7)	0.45 (0.21–0.98)
≥30	38 (1.9)	355 (1.8)	0.91 (0.58–1.42)	36 (25.4)	330 (24.9)	0.68 (0.39–1.17)	27 (21.6)	112 (13.4)	0.91 (0.46–1.80)
p-value for trend			0.673			0.212			0.664
**Insulin**									
0	2,006 (99.0)	19,964 (98.5)	1.00 (referent)	122 (85.9)	1,114 (83.9)	1.00 (referent)	112 (89.6)	763 (91.2)	1.00 (referent)
1–9	3 (0.2)	62 (0.3)	0.41 (0.13–1.36)	3 (2.1)	44 (3.3)	0.66 (0.20–2.24)	3 (2.4)	20 (2.4)	1.11 (0.30–4.19)
10–29	7 (0.4)	76 (0.4)	0.84 (0.38–1.89)	7 (4.9)	50 (3.8)	1.00 (0.41–2.50)	4 (3.2)	17 (2.0)	1.39 (0.35–5.48)
≥30	11 (0.5)	167 (0.8)	0.62 (0.33–1.17)	10 (7.0)	120 (9.0)	0.56 (0.25–1.26)	6 (4.8)	37 (4.4)	0.58 (0.18–1.83)
p-value for trend			0.147			0.171			0.391

^a^Matched on age, sex, general practice, and number of years of active history in the database, and adjusted for BMI, smoking, MI, arterial hypertension, estrogen use, and all antidiabetics used by the study population.

We found no clear association between sulfonylureas and meningioma, even when we restricted the analysis to patients with diabetes and matched them on duration of diabetes and HbA1c level (p-value for trend = 0.664). By comparison, the number of insulin prescriptions showed a statistically non-significant inverse relation to meningioma (p-value for trend = 0.147). When we matched on duration of diabetes and HbA1c level, the trend was attenuated (p-value for trend = 0.391). This held true for both men (p-value for trend = 0.759) and women (p-value for trend = 0.359). Results did not change materially when BMI was not adjusted for, although 52.2% of long-term users of metformin had a BMI ≥30 kg/m^2^.

## Discussion

Our matched case-control analysis revealed a suggestive inverse association of diabetes with meningioma, which was driven by an inverse relation among women, in whom we also noted a suggestive inverse association with duration of diabetes.

In prior investigations, positive [8, 9], null [10], and statistically non-significant [12] or significant inverse associations [11] were observed between diabetes and the risk of meningioma. A German case-control study based on 306 meningioma patients found a positive association with diabetes that was restricted to certain age- and gender groups (50–69 years for men, 40–49 years and 60–69 years for women) (ORs ranging from 4.30 to 13.94 with p-values ranging from 0.001 to 0.05), but that study did not adjust for possible confounding factors such as BMI or arterial hypertension [8]. A Swedish study [9] including 4,193 meningioma patients differed from our study in that the diagnosis of diabetes was based on hospital discharge letters, which results in the detection of more severe and long-lasting cases of diabetes. In that study, the OR for meningioma in diabetic men increased beginning 6 to 7 years before brain tumor diagnosis, but in diabetic women, as in our study, the ORs for meningioma initially declined (p-value for trend = 0.02) until 1 to 2 years before meningioma diagnosis. A recent cohort study based on 296 meningioma patients derived from the Apolipoprotein MOrtality RISk (AMORIS) cohort found that diabetes was inversely related to meningioma for both sexes combined (HR = 0.45; 95%CI = 0.29–0.71) and the authors specifically discussed the possibility that the decreased risk of meningioma in diabetic patients may be attributed to metformin use, which they did not evaluate in their study, and they were also not able to stratify by sex due to small numbers [11]. A large hospital-based case-control study also found an inverse association between diabetes and risk of meningioma, but results did not reach statistical significance (OR = 0.67; 95%CI = 0.37–1.20), whereas an international population-based case-control study showed no clear relation of diabetes to meningioma [10].

One prior study investigating risk of meningioma in relation to fasting serum glucose levels found no association between the two [7], whereas another study found an inverse relation of fasting serum glucose to meningioma risk in women [11], but both those investigations differed from our study since we investigated HbA1c and not fasting serum glucose levels.

One possible explanation for the inverse association between diabetes and meningioma in women observed in our study is that diabetic women partly suffer from a reduced ability to convert androgens to estrogens in the ovaries [22], and female sex hormones are proposed to increase the risk of meningioma [3, 4]. This hypothesis is supported by the fact that the inverse association between diabetes and risk of meningioma was lost in women taking exogenous estrogens. Because diabetes and antidiabetic treatment are closely interrelated, it is challenging to examine the risk of meningioma in relation to diabetes *per se*, without considering the use of antidiabetic drugs. Therefore, in our main analyses we focused on antidiabetic medications but we conducted important additional analyses that accounted for duration of diabetes and HbA1c level.

Use of sulfonylureas showed no clear association with meningioma, whereas for insulin there was the suggestion of an inverse relation, in particular, when comparing a high vs. low number of prescriptions. For metformin, there was an unexpected borderline statistically significant positive association with meningioma risk in analyses matched on duration of diabetes and level of glycemic control, which was rendered statistically significant in the test for trend restricted to women. Possibly, metformin use leads to hormonal changes in women, such as reduction of luteinizing or follicle stimulation hormone as observed in polycystic ovary syndrome [23], which may influence free estradiol levels and thereby risk of meningioma. In addition, although we adjusted our analyses for BMI, we cannot fully exclude residual confounding by adiposity. Metformin is the first-line treatment for obese type-II diabetic patients, which may lead to a higher proportion of obese patients taking metformin [24].

The fact that metformin inhibits mTOR in experimental models [15] but shows no inhibitory effects on meningioma development, where mTOR signalling plays an important role [16, 17], may be explained by several factors. Antidiabetic doses of metformin may not be sufficient to inhibit mTOR in meningioma development, though metformin passes the blood-brain barrier [25]. Consistent with this hypothesis, meningioma cells treated with clinically relevant doses of metformin were not significantly inhibited *in vitro* [18]. Also, even though mTOR signalling is an important pathogenic factor for established meningiomas, its blockage might not inhibit initial meningioma development. Additionally, the sample size of diabetic meningioma patients taking metformin in our study may not have been sufficient to detect significant results.

Certain potential shortcomings of our study need to be discussed. Data on ionizing radiation, the only known modifiable risk factor for meningioma [26], were not available in the CPRD. However, the proportion of meningioma cases due to radiation is small, and patients with cancers other than non-melanoma skin cancer were excluded from the study population. Another limitation is missing information on molecular subtype or degree of malignancy of the investigated meningiomas. Socioeconomic status, education level, and lifestyle factors [5, 27] were not taken into account in our analyses due to limited information on these variables, but may influence meningioma risk. Also, we may have under-ascertained patients with meningioma diagnosis due to subclinical meningiomas [28], but the number of undiagnosed meningioma patients should not differ substantially between cases and controls, especially after shifting the index date back in time by three years. Additionally, although shifting the index date backwards in time by three years, we may have failed to encompass the true latency period of meningiomas due to their slow growing behaviour. Finally, we cannot fully rule out confounding by indication. For example, metformin may have been used in less severe cases of diabetes compared to insulin [29]. Hence, our analyses of antidiabetic drugs in relation to risk of meningioma were adjusted for duration of diabetes and HbA1c level to account for differences in diabetes severity.

Our study has a number of notable strengths. To the best of our knowledge, the current study is the first to comprehensively evaluate the relations of diabetes, duration of diabetes, level of glycemic control, and antidiabetic drug use to risk of meningioma. Additionally, we performed a number of sensitivity analyses, such as matching on duration of diabetes and level of glycemic control. The CPRD is a well-validated and large database [20]. Cases and controls were generated from a pre-existing database, which minimized selection bias. Further, there was no recall bias because the data on medications and concomitant diseases were collected prospectively and without a pre-specified study hypothesis. We shifted the index date back in time by three years to account for various potential biases. Finally, we excluded patients with less than three years of active history in the CPRD before the index date in order to increase the chance of including incident meningioma cases.

In summary, diabetes was inversely related to risk of meningioma among women, whereas increasing use of metformin was associated with increased risk of meningioma among women. Our study does not provide evidence for a protective association of metformin use to meningioma risk. Further research is however needed to evaluate whether metformin use is potentially associated with improved survival of meningioma patients.

## Supporting information

S1 Table READ codes for meningioma used in this study and corresponding descriptions. (DOCX)
