# Supplementary material for: Diabetes, use of metformin, and the risk of meningioma
Source: PLoS One. 2017 Jul 14;12(7):e0181089. doi: 10.1371/journal.pone.0181089 (PMC5510861; doi:10.1371/journal.pone.0181089)
Supplement: S1 Table — (DOCX) [file pone.0181089.s001.docx]

**S1 Table. READ codes for meningioma used in this study and corresponding descriptions**

| READ code and description |
| --- |

B7F2000 Cerebral meningioma

B7F4000 Spinal meningioma

BBd..00 [M]Meningiomas

BBd0.00 [M]Meningioma NOS

BBd1.00 [M]Meningiomatosis NOS

BBd1.11 [M]Diffuse meningiomatosis

BBd1.12 [M]Multiple meningiomatosis

BBd2.00 [M]Meningioma; malignant

BBd3.00 [M]Meningotheliomatous meningioma

BBd3.11 [M]Endotheliomatous meningioma

BBd3.12 [M]Syncytial meningioma

BBd4.00 [M]Fibrous meningioma

BBd5.00 [M]Psammomatous meningioma

BBd6.00 [M]Angiomatous meningioma

BBd7.00 [M]Haemangioblastic meningioma

BBd7.11 [M]Angioblastic meningioma

BBd8.00 [M]Haemangiopericytic meningioma

BBd9.00 [M]Transitional meningioma

BBd9.11 [M]Mixed meningioma

BBdA.00 [M]Papillary meningioma

BBdz.00 [M]Meningioma NOS
